# Supplementary figures and images for: Use of minimally invasive tissue sampling to determine the contribution of diarrheal diseases to under-five mortality and associated co-morbidities and co-infections in children with fatal diarrheal diseases in Africa and Bangladesh
Source: PLOS Glob Public Health. 2025 Jun 25;5(6):e0004772. doi: 10.1371/journal.pgph.0004772 (PMC12193650; doi:10.1371/journal.pgph.0004772)

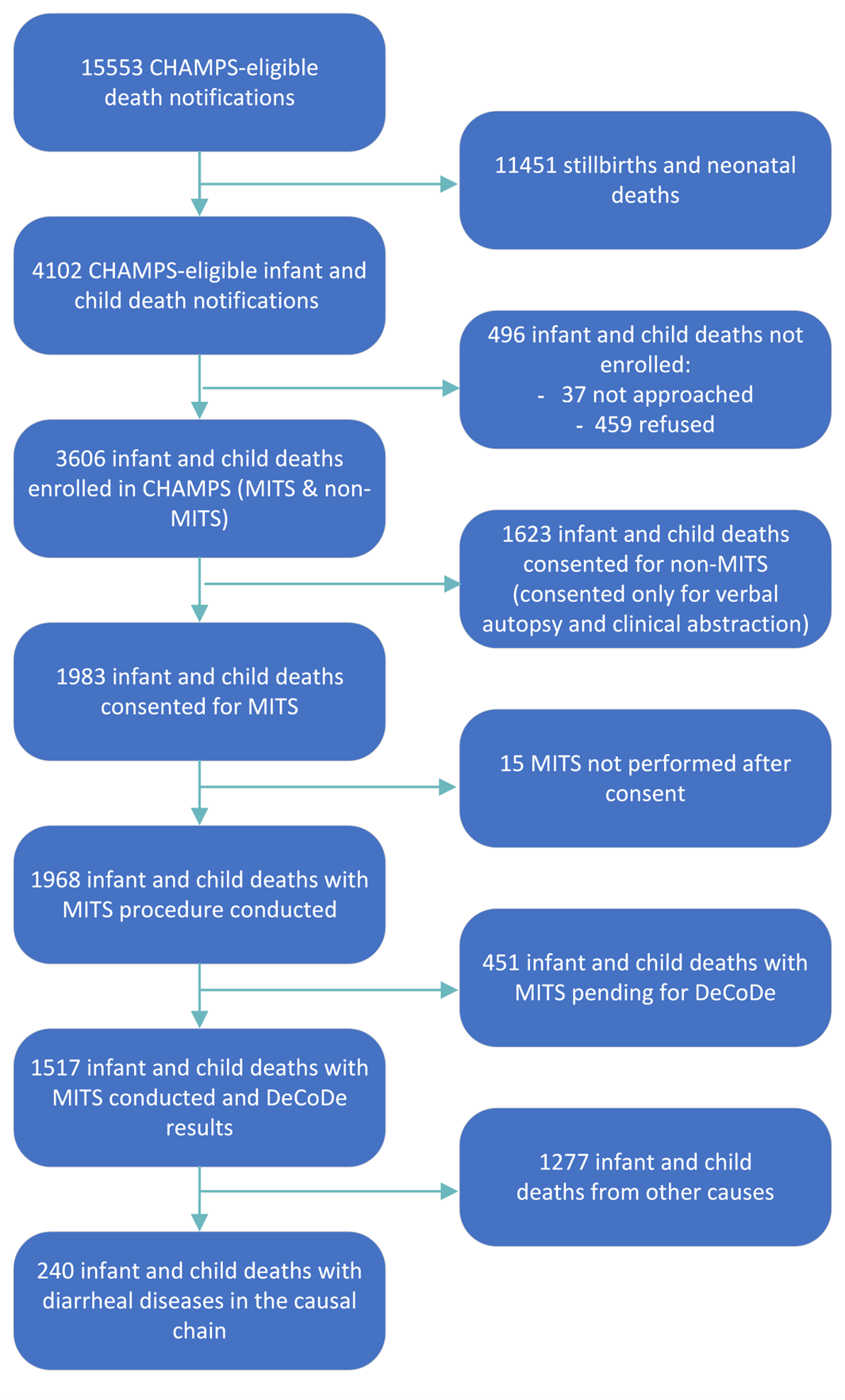

Supplement: S1 Fig — (PNG) [file pgph.0004772.s001.png]

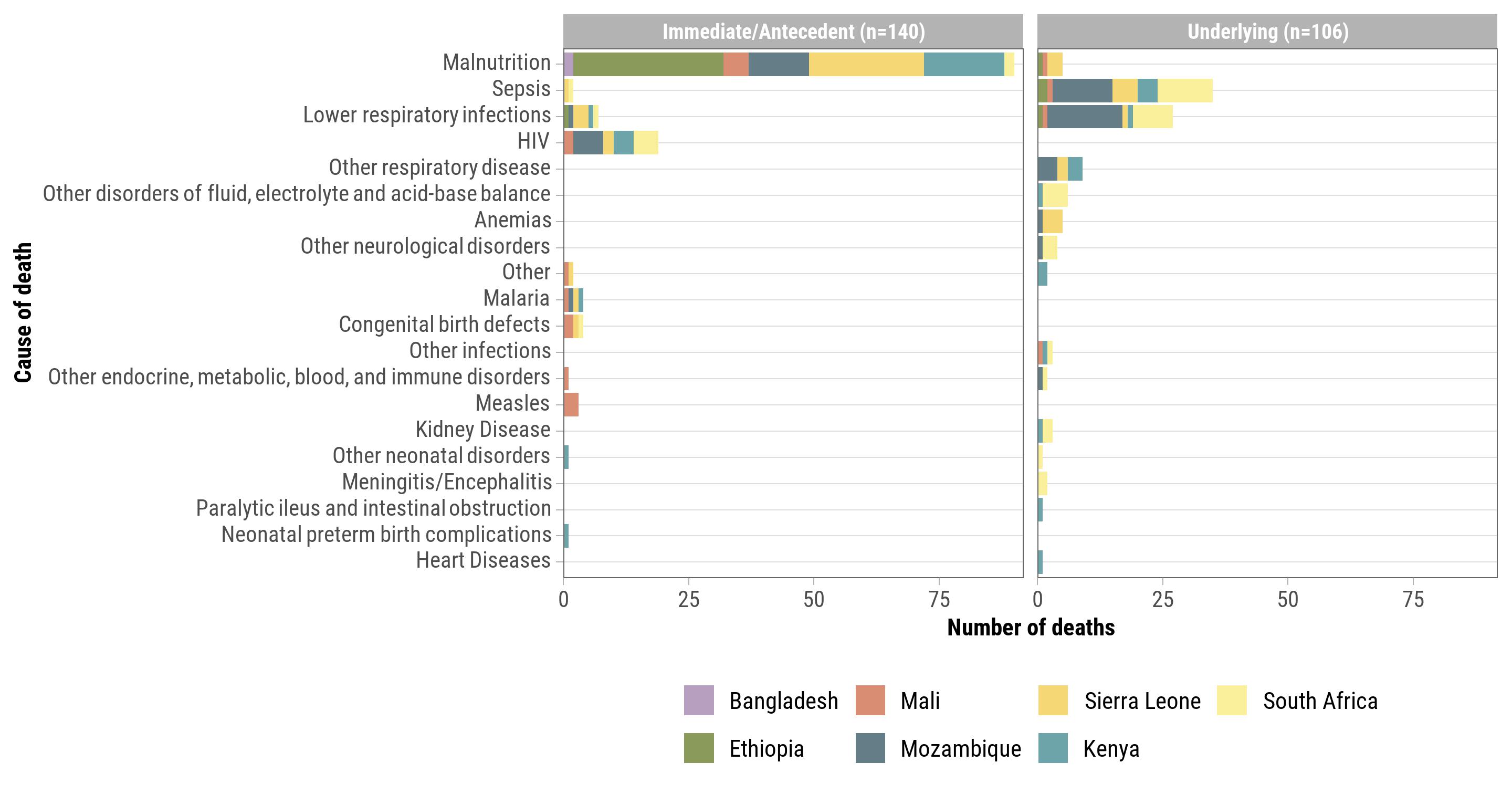

Supplement: S2 Fig — There were 240 deaths with diarrheal diseases in the causal chain: six deaths had diarrheal diseases listed as both underlying and immediate causes of death. (JPG) [file pgph.0004772.s002.jpg]

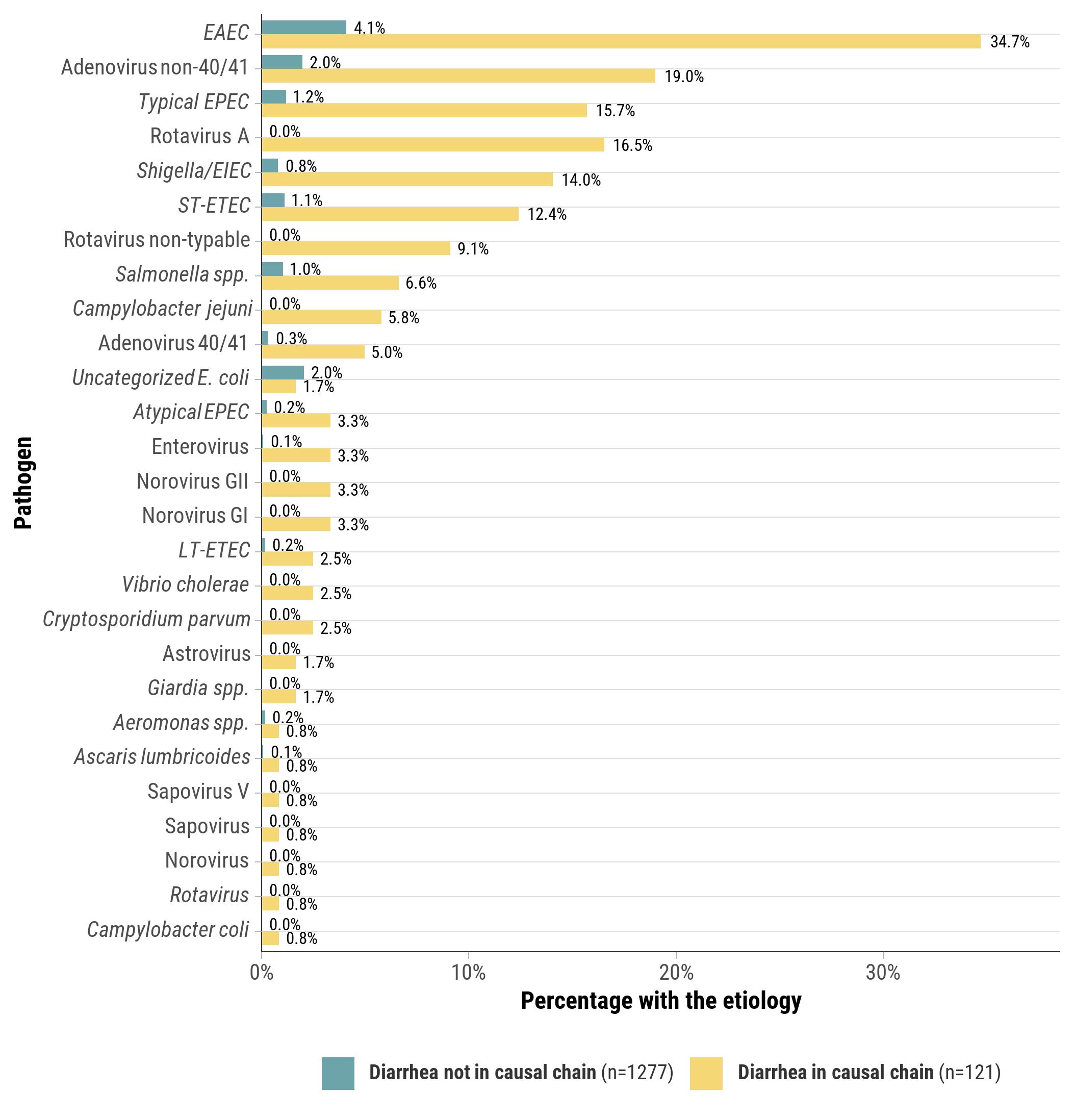

Supplement: S4 Fig — (JPG) [file pgph.0004772.s004.jpg]
