# Supplementary material for: Use of minimally invasive tissue sampling to determine the contribution of diarrheal diseases to under-five mortality and associated co-morbidities and co-infections in children with fatal diarrheal diseases in Africa and Bangladesh
Source: PLOS Glob Public Health. 2025 Jun 25;5(6):e0004772. doi: 10.1371/journal.pgph.0004772 (PMC12193650; doi:10.1371/journal.pgph.0004772)
Supplement: S3 Fig — (JPG) [file pgph.0004772.s003.docx]

| **S3 Table**. Pathogens attributed to diarrheal disease in the causal chain by age group, CHAMPS Network, 2016–2023. | | | |
| --- | --- | --- | --- |
| Pathogen | Total  (N = 240) | Infant  (N = 135) | Child  (N = 105) |
| *EAEC* | 42 (17.5) | 24 (17.8) | 18 (17.1) |
| Adenovirus non-40/41 | 23 (9.6) | 10 (7.4) | 13 (12.4) |
| Rotavirus A | 20 (8.3) | 14 (10.4) | 6 (5.7) |
| *Typical EPEC* | 19 (7.9) | 9 (6.7) | 10 (9.5) |
| *Shigella/EIEC* | 17 (7.1) | 5 (3.7) | 12 (11.4) |
| *ST-ETEC* | 15 (6.2) | 6 (4.4) | 9 (8.6) |
| Rotavirus non-typable | 12 (5.0) | 9 (6.7) | 3 (2.9) |
| *Salmonella spp.* | 8 (3.3) | 2 (1.5) | 6 (5.7) |
| *Campylobacter jejuni* | 7 (2.9) | 6 (4.4) | 1 (1.0) |
| Adenovirus 40/41 | 6 (2.5) | 1 (0.7) | 5 (4.8) |
| *Atypical EPEC* | 4 (1.7) | 3 (2.2) | 1 (1.0) |
| Enterovirus | 4 (1.7) | 4 (3.0) | 0 (0.0) |
| Norovirus GI | 4 (1.7) | 2 (1.5) | 2 (1.9) |
| Norovirus GII | 4 (1.7) | 4 (3.0) | 0 (0.0) |
| *Cryptosporidium parvum* | 3 (1.2) | 1 (0.7) | 2 (1.9) |
| *LT-ETEC* | 3 (1.2) | 2 (1.5) | 1 (1.0) |
| *Vibrio cholerae* | 3 (1.2) | 1 (0.7) | 2 (1.9) |
| Astrovirus | 2 (0.8) | 1 (0.7) | 1 (1.0) |
| *Giardia spp.* | 2 (0.8) | 1 (0.7) | 1 (1.0) |
| *Aeromonas spp.* | 1 (0.4) | 0 (0.0) | 1 (1.0) |
| *Ascaris lumbricoides* | 1 (0.4) | 1 (0.7) | 0 (0.0) |
| *Campylobacter coli* | 1 (0.4) | 1 (0.7) | 0 (0.0) |
| Norovirus | 1 (0.4) | 0 (0.0) | 1 (1.0) |
| Sapovirus | 1 (0.4) | 1 (0.7) | 0 (0.0) |
| Sapovirus V | 1 (0.4) | 1 (0.7) | 0 (0.0) |
