# Supplementary material for: Use of minimally invasive tissue sampling to determine the contribution of diarrheal diseases to under-five mortality and associated co-morbidities and co-infections in children with fatal diarrheal diseases in Africa and Bangladesh
Source: PLOS Glob Public Health. 2025 Jun 25;5(6):e0004772. doi: 10.1371/journal.pgph.0004772 (PMC12193650; doi:10.1371/journal.pgph.0004772)
Supplement: S1 Table — (DOCX) [file pgph.0004772.s005.docx]

**S1 Table**. Rotavirus vaccination status for infant and child deaths, CHAMPS Network, 2016–2023 (N=1517).

|  | **Total**  N=1517 | **South Africa**  N=339 | **Kenya**  N=366 | **Sierra Leone**  N=358 | **Mozambique**  N=258 | **Mali**  N=103 | **Ethiopia**  N=83 | **Bangladesh**  N=10 |
| --- | --- | --- | --- | --- | --- | --- | --- | --- |
| **Rotavirus vaccination status** |  |  |  |  |  |  |  |  |
| **All deaths** |  |  |  |  |  |  |  |  |
| Vaccinated | 322 (21.2) | 5 (1.5) | 150 (41.0) | 96 (26.8) | 26 (10.1) | 45 (43.7) | 0 (0) | 0 (0) |
| Unvaccinated or unknown | 1195 (78.8) | 334 (98.5) | 216 (59.0) | 262 (73.2) | 232 (89.9) | 58 (56.3) | 83 (100.0) | 10 (100.0) |
| **Diarrhea is cause of death (N = 240)** |  |  |  |  |  |  |  |  |
| Vaccinated | 50 (20.8) | 0 (0) | 23 (45.1) | 13 (28.3) | 6 (10.7) | 8 (44.4) | 0 (0) | 0 (0) |
| Unvaccinated or unknown | 190 (79.2) | 32 (100.0) | 28 (54.9) | 33 (71.7) | 50 (89.3) | 10 (55.6) | 34 (100.0) | 3 (100.0) |
| **Rotavirus in causal chain (N = 32)** |  |  |  |  |  |  |  |  |
| Vaccinated | 8 (25.0)^a^ | 0 (0) | 3 (37.5) | 2 (22.2) | 2 (28.6) | 1 (100.0) | 0 (0) | 0 (0) |
| Unvaccinated or unknown | 24 (75.0) | 7 (100.0) | 5 (62.5) | 7 (77.8) | 5 (71.4) | 0 (0) | 0 (0) | 0 (0) |
| Median days between second rotavirus vaccine dose and death for these eight deaths was 219 days (IQR: 138, 383). | | | | | | | | |
