# Supplementary material for: Use of minimally invasive tissue sampling to determine the contribution of diarrheal diseases to under-five mortality and associated co-morbidities and co-infections in children with fatal diarrheal diseases in Africa and Bangladesh
Source: PLOS Glob Public Health. 2025 Jun 25;5(6):e0004772. doi: 10.1371/journal.pgph.0004772 (PMC12193650; doi:10.1371/journal.pgph.0004772)
Supplement: S2 Table — (DOCX) [file pgph.0004772.s006.docx]

**S2 Table**. Other causes of death for infants (1 to <12 months) and children (12-59 months) in the causal chain for deaths with diarrheal disease as the underlying cause (N=106)^a^ by site, CHAMPS Network, 2016–2023.

|  | **Total** | | | **South Africa** | | | **Kenya** | | | **Sierra Leone** | | | **Mozambique** | | | **Mali** | | | **Ethiopia** | | |
| --- | --- | --- | --- | --- | --- | --- | --- | --- | --- | --- | --- | --- | --- | --- | --- | --- | --- | --- | --- | --- | --- |
|  | N=106 | N=71 | N=35 | N=22 | N=18 | N=4 | N=27 | N=20 | N=7 | N=14 | N=12 | N=2 | N=36 | N=16 | N=20 | N=3 | N=1 | N=2 | N=3 | N=3 | N=0 |
|  | Total | 1-11m | 12-59m | Total | 1-11m | 12-59m | Total | 1-11m | 12-59m | Total | 1-11m | 12-59m | Total | 1-11m | 12-59m | Total | 1-11m | 12-59m | Total | 1-11m | 12-59m |
| Sepsis | 35 (33.0) | 22 (31.0) | 13 (37.1) | 11 (50.0) | 10 (55.6) | 1 (25.0) | 4 (14.8) | 2 (10.0) | 2 (28.6) | 5 (35.7) | 3 (25.0) | 2 (100.0) | 12 (33.3) | 5 (31.2) | 7 (35.0) | 1 (33.3) | 0 (0) | 1 (50.0) | 2 (66.7) | 2 (66.7) | 0 (0) |
| Lower respiratory infections | 27 (25.5) | 16 (22.5) | 11 (31.4) | 8 (36.4) | 7 (38.9) | 1 (25.0) | 1 (3.7) | 1 (5.0) | 0 (0) | 1 (7.1) | 0 (0) | 1 (50.0) | 15 (41.7) | 7 (43.8) | 8 (40.0) | 1 (33.3) | 0 (0) | 1 (50.0) | 1 (33.3) | 1 (33.3) | 0 (0) |
| Other respiratory disease | 9 (8.5) | 6 (8.5) | 3 (8.6) | 0 (0) | 0 (0) | 0 (0) | 3 (11.1) | 2 (10.0) | 1 (14.3) | 2 (14.3) | 2 (16.7) | 0 (0) | 4 (11.1) | 2 (12.5) | 2 (10.0) | 0 (0) | 0 (0) | 0 (0) | 0 (0) | 0 (0) | 0 (0) |
| Anemias | 5 (4.7) | 2 (2.8) | 3 (8.6) | 0 (0) | 0 (0) | 0 (0) | 0 (0) | 0 (0) | 0 (0) | 4 (28.6) | 2 (16.7) | 2 (100.0) | 1 (2.8) | 0 (0) | 1 (5.0) | 0 (0) | 0 (0) | 0 (0) | 0 (0) | 0 (0) | 0 (0) |
| Malnutrition | 5 (4.7) | 3 (4.2) | 2 (5.7) | 0 (0) | 0 (0) | 0 (0) | 0 (0) | 0 (0) | 0 (0) | 3 (21.4) | 2 (16.7) | 1 (50.0) | 0 (0) | 0 (0) | 0 (0) | 1 (33.3) | 0 (0) | 1 (50.0) | 1 (33.3) | 1 (33.3) | 0 (0) |
| Other disorders of fluid, electrolyte and acid-base balance | 6 (5.7) | 6 (8.5) | 0 (0) | 5 (22.7) | 5 (27.8) | 0 (0) | 1 (3.7) | 1 (5.0) | 0 (0) | 0 (0) | 0 (0) | 0 (0) | 0 (0) | 0 (0) | 0 (0) | 0 (0) | 0 (0) | 0 (0) | 0 (0) | 0 (0) | 0 (0) |
| Other neurological disorders | 4 (3.8) | 3 (4.2) | 1 (2.9) | 3 (13.6) | 3 (16.7) | 0 (0) | 0 (0) | 0 (0) | 0 (0) | 0 (0) | 0 (0) | 0 (0) | 1 (2.8) | 0 (0) | 1 (5.0) | 0 (0) | 0 (0) | 0 (0) | 0 (0) | 0 (0) | 0 (0) |
| Kidney Disease | 3 (2.8) | 3 (4.2) | 0 (0) | 2 (9.1) | 2 (11.1) | 0 (0) | 1 (3.7) | 1 (5.0) | 0 (0) | 0 (0) | 0 (0) | 0 (0) | 0 (0) | 0 (0) | 0 (0) | 0 (0) | 0 (0) | 0 (0) | 0 (0) | 0 (0) | 0 (0) |
| Meningitis/Encephalitis | 2 (1.9) | 1 (1.4) | 1 (2.9) | 2 (9.1) | 1 (5.6) | 1 (25.0) | 0 (0) | 0 (0) | 0 (0) | 0 (0) | 0 (0) | 0 (0) | 0 (0) | 0 (0) | 0 (0) | 0 (0) | 0 (0) | 0 (0) | 0 (0) | 0 (0) | 0 (0) |
| Other endocrine, metabolic, blood, and immune disorders | 2 (1.9) | 1 (1.4) | 1 (2.9) | 1 (4.5) | 1 (5.6) | 0 (0) | 0 (0) | 0 (0) | 0 (0) | 0 (0) | 0 (0) | 0 (0) | 1 (2.8) | 0 (0) | 1 (5.0) | 0 (0) | 0 (0) | 0 (0) | 0 (0) | 0 (0) | 0 (0) |
| Other infections | 3 (2.8) | 0 (0) | 3 (8.6) | 1 (4.5) | 0 (0) | 1 (25.0) | 1 (3.7) | 0 (0) | 1 (14.3) | 0 (0) | 0 (0) | 0 (0) | 0 (0) | 0 (0) | 0 (0) | 1 (33.3) | 0 (0) | 1 (50.0) | 0 (0) | 0 (0) | 0 (0) |
| Other | 2 (1.9) | 2 (2.8) | 0 (0) | 0 (0) | 0 (0) | 0 (0) | 2 (7.4) | 2 (10.0) | 0 (0) | 0 (0) | 0 (0) | 0 (0) | 0 (0) | 0 (0) | 0 (0) | 0 (0) | 0 (0) | 0 (0) | 0 (0) | 0 (0) | 0 (0) |
| Heart Diseases | 1 (0.9) | 1 (1.4) | 0 (0) | 0 (0) | 0 (0) | 0 (0) | 1 (3.7) | 1 (5.0) | 0 (0) | 0 (0) | 0 (0) | 0 (0) | 0 (0) | 0 (0) | 0 (0) | 0 (0) | 0 (0) | 0 (0) | 0 (0) | 0 (0) | 0 (0) |
| Other neonatal disorders | 1 (0.9) | 1 (1.4) | 0 (0) | 1 (4.5) | 1 (5.6) | 0 (0) | 0 (0) | 0 (0) | 0 (0) | 0 (0) | 0 (0) | 0 (0) | 0 (0) | 0 (0) | 0 (0) | 0 (0) | 0 (0) | 0 (0) | 0 (0) | 0 (0) | 0 (0) |
| Paralytic ileus and intestinal obstruction | 1 (0.9) | 1 (1.4) | 0 (0) | 0 (0) | 0 (0) | 0 (0) | 1 (3.7) | 1 (5.0) | 0 (0) | 0 (0) | 0 (0) | 0 (0) | 0 (0) | 0 (0) | 0 (0) | 0 (0) | 0 (0) | 0 (0) | 0 (0) | 0 (0) | 0 (0) |

^a^ Excludes one infant death from Bangladesh with diarrheal diseases as the underlying cause of death, which did not have any other causes of death in the causal chain.
