# Supplementary material for: Use of minimally invasive tissue sampling to determine the contribution of diarrheal diseases to under-five mortality and associated co-morbidities and co-infections in children with fatal diarrheal diseases in Africa and Bangladesh
Source: PLOS Glob Public Health. 2025 Jun 25;5(6):e0004772. doi: 10.1371/journal.pgph.0004772 (PMC12193650; doi:10.1371/journal.pgph.0004772)
Supplement: S4 Table — (DOCX) [file pgph.0004772.s008.docx]

| **S4 Table**. Pathogens attributed to diarrheal disease in the causal chain for infant and child deaths with and without malnutrition in causal chain or as other significant condition, CHAMPS Network, 2016–2023. | | |
| --- | --- | --- |
| Pathogen | Malnutrition  (N = 135) | No Malnutrition  (N = 105) |
| EAEC | 28 (20.7) | 14 (13.3) |
| Adenovirus non-40/41 | 11 (8.1) | 12 (11.4) |
| Rotavirus A | 7 (5.2) | 13 (12.4) |
| Typical EPEC | 14 (10.4) | 5 (4.8) |
| Shigella/EIEC | 11 (8.1) | 6 (5.7) |
| ST-ETEC | 14 (10.4) | 1 (1.0) |
| Rotavirus non-typable | 4 (3.0) | 8 (7.6) |
| *Salmonella spp.* | 6 (4.4) | 2 (1.9) |
| *Campylobacter jejuni* | 2 (1.5) | 5 (4.8) |
| Adenovirus 40/41 | 1 (0.7) | 5 (4.8) |
| Atypical EPEC | 2 (1.5) | 2 (1.9) |
| Enterovirus | 3 (2.2) | 1 (1.0) |
| Norovirus GI | 3 (2.2) | 1 (1.0) |
| Norovirus GII | 2 (1.5) | 2 (1.9) |
| *Cryptosporidium parvum* | 2 (1.5) | 1 (1.0) |
| LT-ETEC | 2 (1.5) | 1 (1.0) |
| *Vibrio cholerae* | 1 (0.7) | 2 (1.9) |
| *Aeromonas spp.* | 1 (0.7) | 0 (0.0) |
| *Ascaris lumbricoides* | 1 (0.7) | 0 (0.0) |
| Astrovirus | 1 (0.7) | 1 (1.0) |
| *Campylobacter coli* | 1 (0.7) | 0 (0.0) |
| *Giardia spp.* | 1 (0.7) | 1 (1.0) |
| Norovirus | 0 (0.0) | 1 (1.0) |
| Sapovirus | 0 (0.0) | 1 (1.0) |
| Sapovirus V | 1 (0.7) | 0 (0.0) |
