# Supplementary material for: Use of minimally invasive tissue sampling to determine the contribution of diarrheal diseases to under-five mortality and associated co-morbidities and co-infections in children with fatal diarrheal diseases in Africa and Bangladesh
Source: PLOS Glob Public Health. 2025 Jun 25;5(6):e0004772. doi: 10.1371/journal.pgph.0004772 (PMC12193650; doi:10.1371/journal.pgph.0004772)
Supplement: S5 Table — (DOCX) [file pgph.0004772.s009.docx]

| **S5 Table.** Pathogens attributed to diarrheal disease among infant and child deaths, stratified by nutritional and HIV status, CHAMPS Network, 2016–2023. | | | |
| --- | --- | --- | --- |
| Pathogen | Malnutrition  (N = 135) | HIV Positive  (N = 20) | Neither Malnutrition nor HIV  (N = 98) |
| EAEC | 28 (20.7) | 1 (5.0) | 13 (13.3) |
| Adenovirus non-40/41 | 11 (8.1) | 0 (0.0) | 12 (12.2) |
| Rotavirus A | 7 (5.2) | 0 (0.0) | 13 (13.3) |
| Typical EPEC | 14 (10.4) | 1 (5.0) | 5 (5.1) |
| Shigella/EIEC | 11 (8.1) | 0 (0.0) | 6 (6.1) |
| ST-ETEC | 14 (10.4) | 0 (0.0) | 1 (1.0) |
| Rotavirus non-typable | 4 (3.0) | 1 (5.0) | 7 (7.1) |
| *Salmonella spp.* | 6 (4.4) | 0 (0.0) | 2 (2.0) |
| *Campylobacter jejuni* | 2 (1.5) | 1 (5.0) | 5 (5.1) |
| Adenovirus 40/41 | 1 (0.7) | 2 (10.0) | 4 (4.1) |
| Atypical EPEC | 2 (1.5) | 0 (0.0) | 2 (2.0) |
| Enterovirus | 3 (2.2) | 0 (0.0) | 1 (1.0) |
| Norovirus GI | 3 (2.2) | 0 (0.0) | 1 (1.0) |
| Norovirus GII | 2 (1.5) | 0 (0.0) | 2 (2.0) |
| *Cryptosporidium parvum* | 2 (1.5) | 0 (0.0) | 1 (1.0) |
| LT-ETEC | 2 (1.5) | 0 (0.0) | 1 (1.0) |
| *Vibrio cholerae* | 1 (0.7) | 0 (0.0) | 2 (2.0) |
| *Aeromonas spp.* | 1 (0.7) | 0 (0.0) | 0 (0.0) |
| *Ascaris lumbricoides* | 1 (0.7) | 0 (0.0) | 0 (0.0) |
| Astrovirus | 1 (0.7) | 1 (5.0) | 1 (1.0) |
| *Campylobacter coli* | 1 (0.7) | 0 (0.0) | 0 (0.0) |
| *Giardia spp.* | 1 (0.7) | 0 (0.0) | 1 (1.0) |
| Norovirus | 0 (0.0) | 0 (0.0) | 1 (1.0) |
| Sapovirus | 0 (0.0) | 0 (0.0) | 1 (1.0) |
| Sapovirus V | 1 (0.7) | 0 (0.0) | 0 (0.0) |
| This table shows the number and percentage of diarrhea-attributed deaths in which each pathogen was identified in the causal chain, stratified by (1) presence of malnutrition (in the causal chain or as an “other significant” condition), (2) HIV-infected status (regardless of causal chain attribution), and (3) absence of both malnutrition and HIV infection. | | | |
