# Supplementary material for: Use of minimally invasive tissue sampling to determine the contribution of diarrheal diseases to under-five mortality and associated co-morbidities and co-infections in children with fatal diarrheal diseases in Africa and Bangladesh
Source: PLOS Glob Public Health. 2025 Jun 25;5(6):e0004772. doi: 10.1371/journal.pgph.0004772 (PMC12193650; doi:10.1371/journal.pgph.0004772)
Supplement: S6 Table — (DOCX) [file pgph.0004772.s010.docx]

| **S6 Table. Crude and adjusted total under-five mortality fractions and 90% Bayesian credible intervals due to diarrheal diseases at all sites and catchments within the CHAMPS Network.** | | | | | | | | | | | | | | | | | | | | | | | | | | | | | | | | | |
| --- | --- | --- | --- | --- | --- | --- | --- | --- | --- | --- | --- | --- | --- | --- | --- | --- | --- | --- | --- | --- | --- | --- | --- | --- | --- | --- | --- | --- | --- | --- | --- | --- | --- |
| **Sites and Catchments** | **Years** | **Underlying** | | | | | | | | | | | | | | | **Causal Chain** | | | | | | | | | | | | | | | | |
|  |  | n | Crude  (per 10,000 births) | | | | | Adjusted  (per 10,000 births) | | | | | | | | n | | | | Crude  (per 10,000 births) | | | | | | Adjusted  (per 10,000 births) | | | | | | |  |
|  |  |  | CSMF | 90% Bayesian CrI | | | CSMF | | | 90% Bayesian CrI | | | Adjusted Factors | |  | | | | CSMF | | | 90% Bayesian CrI | | | CSMF | | | 90% Bayesian CrI | | | Adjusted Factors |  |  |
|  |  |  | Estimate | Lower | Upper | Estimate | | | Lower | | Upper |  | |  | | | | Estimate | | | Lower | | Upper | Estimate | | | Lower | | Upper |  | |  |  |
| **Bangladesh** |  |  |  |  |  |  | | |  | |  |  | |  | | | |  | | |  | |  |  | | |  | |  |  | |  |  |
| Baliakandi & Faridpur | 2017-2020 | 0 | 0.0 | 0.0 | 0.9 | 0.0 | | | 0.0 | | 0.0 | none | | 1 | | | | 0.5 | | | 0.1 | | 1.8 | 5.9 | | | 4.8 | | 7.1 | age | |  |  |
| Baliakandi | 2017-2020 | 0 | 0.0 | 0.0 | 13.5 | 0.0 | | | 0.0 | | 13.5 | none | | 1 | | | | 1.2 | | | 0.2 | | 4.6 | 7.0 | | | 5.9 | | 8.3 | age | |  |  |
| Faridpur | 2018-2020 | 0 | 0.0 | 0.0 | 1.5 | 0.0 | | | 0.0 | | 1.5 | none | | 0 | | | | 0.0 | | | 0.0 | | 1.5 | 0.0 | | | 0.0 | | 1.5 | none | |  |  |
| **Ethiopia** |  |  |  |  |  |  | | |  | |  |  | |  | | | |  | | |  | |  |  | | |  | |  |  | |  |  |
| Harar, Haramaya & Kersa | 2019-2020 | 1 | 0.6 | 0.1 | 2.3 | 2.7 | | | 2.1 | | 3.5 | age | | 6 | | | | 3.6 | | | 1.8 | | 6.6 | 17.6 | | | 15.9 | | 19.4 | age, location | |  |  |
| Harar | 2019-2020 | 1 | 1.3 | 0.2 | 4.8 | 4.6 | | | 2.3 | | 8.5 | age, location | | 1 | | | | 1.3 | | | 0.2 | | 4.8 | 4.6 | | | 2.3 | | 8.5 | age, location | |  |  |
| Haramaya | 2020-2020 | 0 | 0.0 | 0.0 | 5.6 | 0.0 | | | 0.0 | | 5.6 | none | | 0 | | | | 0.0 | | | 0.0 | | 5.6 | 0.0 | | | 0.0 | | 5.6 | none | |  |  |
| Kersa | 2019-2020 | 0 | 0.0 | 0.0 | 3.5 | 0.0 | | | 0.0 | | 3.5 | none | | 5 | | | | 9.3 | | | 4.3 | | 17.3 | 23.5 | | | 21.6 | | 25.6 | age | |  |  |
| **Kenya** |  |  |  |  |  |  | | |  | |  |  | |  | | | |  | | |  | |  |  | | |  | |  |  | |  |  |
| Manyatta & Siaya | 2017-2020 | 11 | 2.2 | 1.3 | 3.5 | 2.4 | | | 1.9 | | 3.0 | age | | 25 | | | | 5.1 | | | 3.6 | | 6.9 | 5.7 | | | 4.9 | | 6.6 | age | |  |  |
| Manyatta | 2017-2020 | 9 | 3.3 | 1.9 | 5.4 | 3.4 | | | 2.2 | | 5.1 | age | | 20 | | | | 7.3 | | | 5.0 | | 10.2 | 7.6 | | | 5.7 | | 9.9 | age | |  |  |
| Siaya | 2017-2020 | 2 | 18.4 | 5.3 | 50.5 | 28.1 | | | 19.7 | | 38.9 | age | | 5 | | | | 2.3 | | | 1.1 | | 4.5 | 2.3 | | | 1.7 | | 3.0 | age | |  |  |
| **Mali** |  |  |  |  |  |  | | |  | |  |  | |  | | | |  | | |  | |  |  | | |  | |  |  | |  |  |
| Bamako | 2017-2020 | 1 | 0.5 | 0.1 | 2.0 | 0.5 | | | 0.1 | | 2.0 | none | | 9 | | | | 4.7 | | | 2.6 | | 7.7 | 4.7 | | | 2.6 | | 7.7 | none | |  |  |
| **Mozambique** |  |  |  |  |  |  | | |  | |  |  | |  | | | |  | | |  | |  |  | | |  | |  |  | |  |  |
| Manhiça & Quelimane | 2017-2020 | 23 | 4.8 | 3.4 | 6.6 | 7.2 | | | 6.1 | | 8.5 | age | | 28 | | | | 5.9 | | | 4.3 | | 7.8 | 8.9 | | | 7.6 | | 10.3 | age | |  |  |
| Manhiça | 2017-2020 | 14 | 5.0 | 3.2 | 7.6 | 7.2 | | | 6.3 | | 8.1 | age | | 16 | | | | 5.8 | | | 3.8 | | 8.4 | 8.3 | | | 7.4 | | 9.3 | age | |  |  |
| Quelimane | 2019-2020 | 9 | 4.5 | 2.6 | 7.4 | 4.3 | | | 2.7 | | 6.4 | age | | 12 | | | | 6.0 | | | 3.7 | | 9.2 | 5.6 | | | 3.8 | | 8.1 | age, season | |  |  |
| **Sierra Leone** |  |  |  |  |  |  | | |  | |  |  | |  | | | |  | | |  | |  |  | | |  | |  |  | |  |  |
| Makeni | 2018-2020 | 1 | 0.4 | 0.1 | 1.7 | 0.4 | | | 0.1 | | 1.7 | none | | 8 | | | | 3.6 | | | 1.9 | | 6.0 | 3.4 | | | 2.2 | | 4.8 | age | |  |  |
| **South Africa** |  |  |  |  |  |  | | |  | |  |  | |  | | | |  | | |  | |  |  | | |  | |  |  | |  |  |
| Soweto | 2017-2020 | 12 | 1.7 | 1.1 | 2.7 | 1.7 | | | 1.1 | | 2.6 | age, location | | 17 | | | | 2.5 | | | 1.6 | | 3.6 | 2.4 | | | 1.7 | | 3.4 | age, location | |  |  |

*CrI, credible interval; CSMF, cause-specific mortality fraction; VA, verbal autopsy.*

*CSMF includes stillbirths, neonates, infants, and children under five years of age.*
