# Supplementary material for: Use of minimally invasive tissue sampling to determine the contribution of diarrheal diseases to under-five mortality and associated co-morbidities and co-infections in children with fatal diarrheal diseases in Africa and Bangladesh
Source: PLOS Glob Public Health. 2025 Jun 25;5(6):e0004772. doi: 10.1371/journal.pgph.0004772 (PMC12193650; doi:10.1371/journal.pgph.0004772)
Supplement: S7 Table — S5 Table. Minimally invasive tissue sampling (MITS) procedures that were deemed essential for determining causes of death by age group for deaths with diarrheal diseases in the causal chain, CHAMPS Network, 2016–2023. (DOCX) [file pgph.0004772.s011.docx]

| **S7 Table. Crude and adjusted total under-five mortality rates and 90% Bayesian credible intervals due to diarrheal diseases at all sites and catchments within the CHAMPS Network.** | | | | | | | | | | | | | | | | | | | | | | | | | | | | | | |
| --- | --- | --- | --- | --- | --- | --- | --- | --- | --- | --- | --- | --- | --- | --- | --- | --- | --- | --- | --- | --- | --- | --- | --- | --- | --- | --- | --- | --- | --- | --- |
| **Sites and Catchments** | **Years** | **Underlying** | | | | | | | | | | | | **Causal Chain** | | | | | | | | | | | | | | | | |
|  |  | n | Crude  (per 10,000 births) | | | | | Adjusted  (per 10,000 births) | | | | | | n | | | Crude  (per 10,000 births) | | | | | | Adjusted  (per 10,000 births) | | | | | | |  |
|  |  |  | TU5MR | 90% Bayesian CrI | | | TU5MR | | | 90% Bayesian CrI | | | Adjusted Factors |  | | TU5MR | | | 90% Bayesian CrI | | | TU5MR | | | 90% Bayesian CrI | | | Adjusted Factors |  |  |
|  |  |  | Estimate | Lower | Upper | Estimate | | | Lower | | Upper |  | |  | Estimate | | | Lower | | Upper | Estimate | | | Lower | | Upper |  | |  |  |
| **Bangladesh** |  |  |  |  |  |  | | |  | |  |  | |  |  | | |  | |  |  | | |  | |  |  | |  |  |
| Baliakandi & Faridpur | 2017-2020 | 0 | 0.0 | 0.0 | 4.1 | 0.0 | | | 0.0 | | 4.1 | none | | 1 | 2.2 | | | 0.4 | | 8.4 | 26.9 | | | 22.1 | | 32.6 | age | |  |  |
| Baliakandi | 2017-2020 | 0 | 0.0 | 0.0 | 2.3 | 0.0 | | | 0.0 | | 2.3 | none | | 1 | 7.1 | | | 1.3 | | 27.3 | 41.5 | | | 34.7 | | 49.2 | age | |  |  |
| Faridpur | 2018-2020 | 0 | 0.0 | 0.0 | 5.1 | 0.0 | | | 0.0 | | 5.1 | none | | 0 | 0.0 | | | 0.0 | | 5.1 | 0.0 | | | 0.0 | | 5.1 | none | |  |  |
| **Ethiopia** |  |  |  |  |  |  | | |  | |  |  | |  |  | | |  | |  |  | | |  | |  |  | |  |  |
| Harar, Haramaya & Kersa | 2019-2020 | 1 | 7.8 | 1.4 | 30.3 | 35.2 | | | 27.0 | | 45.1 | age | | 6 | 47.1 | | | 23.3 | | 86.2 | 229.0 | | | 207.0 | | 252.0 | age, location | |  |  |
| Harar | 2019-2020 | 1 | 13.3 | 2.4 | 51.0 | 48.4 | | | 23.6 | | 89.2 | age, location | | 1 | 13.3 | | | 2.4 | | 51.0 | 48.4 | | | 23.6 | | 89.2 | age, location | |  |  |
| Haramaya | 2020-2020 | 0 | 0.0 | 0.0 | 17.6 | 0.0 | | | 0.0 | | 17.6 | none | | 0 | 0.0 | | | 0.0 | | 17.6 | 0.0 | | | 0.0 | | 17.6 | none | |  |  |
| Kersa | 2019-2020 | 0 | 0.0 | 0.0 | 63.7 | 0.0 | | | 0.0 | | 63.7 | none | | 5 | 169.0 | | | 79.2 | | 317.0 | 431.0 | | | 395.0 | | 468.0 | age | |  |  |
| **Kenya** |  |  |  |  |  |  | | |  | |  |  | |  |  | | |  | |  |  | | |  | |  |  | |  |  |
| Manyatta & Siaya | 2017-2020 | 11 | 35.6 | 21.2 | 56.4 | 38.1 | | | 30.1 | | 47.7 | age | | 25 | 80.8 | | | 57.9 | | 110.0 | 90.3 | | | 77.7 | | 104.0 | age | |  |  |
| Manyatta | 2017-2020 | 9 | 29.4 | 16.6 | 48.6 | 30.6 | | | 19.8 | | 45.6 | age | | 20 | 65.3 | | | 45.1 | | 91.5 | 68.3 | | | 51.4 | | 88.9 | age | |  |  |
| Siaya | 2017-2020 | 2 | 0.9 | 0.3 | 2.5 | 1.4 | | | 1.0 | | 1.9 | age | | 5 | 46.0 | | | 21.2 | | 89.5 | 46.0 | | | 34.0 | | 61.0 | age | |  |  |
| **Mali** |  |  |  |  |  |  | | |  | |  |  | |  |  | | |  | |  |  | | |  | |  |  | |  |  |
| Bamako | 2017-2020 | 1 | 2.4 | 0.4 | 9.1 | 2.4 | | | 0.4 | | 9.1 | none | | 9 | 21.2 | | | 12.0 | | 35.0 | 21.2 | | | 12.0 | | 35.0 | none | |  |  |
| **Mozambique** |  |  |  |  |  |  | | |  | |  |  | |  |  | | |  | |  |  | | |  | |  |  | |  |  |
| Manhiça & Quelimane | 2017-2020 | 23 | 48.7 | 34.4 | 67.1 | 72.9 | | | 61.3 | | 86.1 | age | | 28 | 59.3 | | | 43.4 | | 79.2 | 89.9 | | | 77.0 | | 104.0 | age | |  |  |
| Manhiça | 2017-2020 | 14 | 51.4 | 32.8 | 77.0 | 73.0 | | | 64.4 | | 82.5 | age | | 16 | 58.7 | | | 38.7 | | 85.7 | 85.1 | | | 75.8 | | 95.2 | age | |  |  |
| Quelimane | 2019-2020 | 9 | 66.6 | 37.8 | 110.0 | 62.9 | | | 39.7 | | 95.2 | age | | 12 | 88.8 | | | 54.6 | | 137.0 | 83.2 | | | 56.0 | | 119.0 | age, season | |  |  |
| **Sierra Leone** |  |  |  |  |  |  | | |  | |  |  | |  |  | | |  | |  |  | | |  | |  |  | |  |  |
| Makeni | 2018-2020 | 1 | 4.3 | 0.8 | 16.8 | 4.3 | | | 0.8 | | 16.8 | none | | 8 | 34.7 | | | 19.0 | | 59.0 | 32.7 | | | 21.9 | | 47.1 | age | |  |  |
| **South Africa** |  |  |  |  |  |  | | |  | |  |  | |  |  | | |  | |  |  | | |  | |  |  | |  |  |
| Soweto | 2017-2020 | 12 | 24.6 | 15.0 | 38.4 | 24.6 | | | 16.0 | | 36.5 | age, location | | 17 | 34.9 | | | 23.1 | | 50.8 | 34.8 | | | 24.3 | | 48.5 | age, location | |  |  |

*CrI, credible interval; TU5MR, total under-five mortality rate; VA, verbal autopsy.*

*TU5MR includes stillbirths, neonates, infants, and children under five years of age.*
