# Supplementary material for: Use of minimally invasive tissue sampling to determine the contribution of diarrheal diseases to under-five mortality and associated co-morbidities and co-infections in children with fatal diarrheal diseases in Africa and Bangladesh
Source: PLOS Glob Public Health. 2025 Jun 25;5(6):e0004772. doi: 10.1371/journal.pgph.0004772 (PMC12193650; doi:10.1371/journal.pgph.0004772)
Supplement: S8 Table — (DOCX) [file pgph.0004772.s012.docx]

| **S8 Table.** Expert (DeCoDe) panel recommendations for preventing diarrheal diseases deaths, CHAMPS Network, 2016–2023. | | | |
| --- | --- | --- | --- |
|  | All | Infants | Children |
|  | N = 214 | N = 119 | N = 95 |
| Improved ANC and obstetric care and management | 7 (3.3) | 6 (5.0) | 1 (1.1) |
| Improved clinical management and quality of care | 148 (69.2) | 79 (66.4) | 69 (72.6) |
| Improved family planning | 12 (5.6) | 5 (4.2) | 7 (7.4) |
| Improved health-seeking behavior | 95 (44.4) | 49 (41.2) | 46 (48.4) |
| Improved health education [immunizations, preventing malnutrition, diarrhea, burns, poisoning, etc.] | 109 (50.9) | 56 (47.1) | 53 (55.8) |
| Improved HIV prevention and control | 17 (7.9) | 9 (7.6) | 8 (8.4) |
| Improved infection prevention and control | 66 (30.8) | 32 (26.9) | 34 (35.8) |
| Improved nutritional support | 77 (36.0) | 36 (30.3) | 41 (43.2) |
| Improved transport system | 12 (5.6) | 6 (5.0) | 6 (6.3) |
| Improved vaccinations | 30 (14.0) | 14 (11.8) | 16 (16.8) |
