# Supplementary material for: Use of minimally invasive tissue sampling to determine the contribution of diarrheal diseases to under-five mortality and associated co-morbidities and co-infections in children with fatal diarrheal diseases in Africa and Bangladesh
Source: PLOS Glob Public Health. 2025 Jun 25;5(6):e0004772. doi: 10.1371/journal.pgph.0004772 (PMC12193650; doi:10.1371/journal.pgph.0004772)
Supplement: S1 Methods — (DOCX) [file pgph.0004772.s013.docx]

**S1 Methods**

*Cause-Specific Mortality Fractions*

Crude cause-specific mortality fractions (cCSMF) were calculated for each catchment area and site by determining the proportion of deaths attributed to diarrheal diseases among all MITS deaths reviewed by the DeCoDe panels. To calculate adjusted cause-specific mortality fractions (aCSMF), factors hypothesized to affect selection (age of the child, sex of the child, location of death, season of death, and verbal autopsy [VA] cause of death) had to meet each of four criteria for inclusion in the adjustment analysis: (1) statistically significantly associated with MITS consent (p<0.10) by chi-square tests; (2) missing <20% data when comparing MITS and non-MITS deaths; (3) statistically significantly associated with diarrheal diseases as the cause of death (p<0.10) by chi-square tests; and (4) missing <20% data when comparing diarrheal and non-diarrheal deaths. One or at most two factors (due to data limitations) were selected for adjustment. CHAMPS deaths (non-MITS and MITS), MITS deaths, and deaths in the target population were stratified by the factors that met selection. Selection probabilities were calculated as the proportion of MITS deaths among all eligible deaths in the target population for each stratum. Due to sparse data, if a stratum had zero diarrheal deaths, it was combined with another stratum with the closest selection probability. Direct standardization was then performed. The target population for most sites was all eligible deaths ascertained in the DSS in each respective catchment area or site.
