# Supplementary material for: Use of minimally invasive tissue sampling to determine the contribution of diarrheal diseases to under-five mortality and associated co-morbidities and co-infections in children with fatal diarrheal diseases in Africa and Bangladesh
Source: PLOS Glob Public Health. 2025 Jun 25;5(6):e0004772. doi: 10.1371/journal.pgph.0004772 (PMC12193650; doi:10.1371/journal.pgph.0004772)
Supplement: Attachment — Submitted filename: MITS CHAMPS_PGPH-D-24-02973.docx [file pgph.0004772.s014.docx]

Abstract:

Line 75: Please specify 'cause'; is this underlying, any, or immediate cause of death?

Intro:

Line 100: This is confusing as 2 causes can not both be the 4th leading cause. As far as I am informed, diarrhoea is the 3rd cause for <5M and malaria the 4th. Please clarfiy and change .

Results:

Line 237: I am surprised by this percentage as we know from the CHAIN data and data from Uganda that close to 60% of all mortality occurs after hospital discharge, at home. Is this high% in your study a potential bias with higher illness severity?

Line 246: Bangladesh seems an outlier in the study; in numbers, %wasted and % underweight. Happy to see this discussed somewhere.

Line 286:

It would be important to know if these children, vaccinated for Rotavirus, where indeed Rota+ve. This then might have been a vaccine failure? Please elaborate and clarify.

Line 290:

This seems an important missing item as in 432 children, information on Rotavirus vaccination is missing. I think that currently 'but rather limitations in accessing complete immunization data', is underestimating this lack of data. Please amend this sentence, showing this.

Line 297:

This is likely a reflection of the fact that many deaths happened in the hospital with sicker children. If you have more information on co-morbidities and signs of illness severity, good to show them. That way, the reader will have a btter understanding of the clinical state these children were in while admitted.

Line 307: You must know if the pathogens/pathogen profile in the groups where diarrhoea is underlying versus immediate is different. It is not unlikely that both groups (also because of the immunodeficiency caused by the malnutrition) leads to different pathogens causing the diarrhoea. Do you have information of children with no pathogen while being malnourished; as in malnutrition-enteropathy leading to death?

Line 307-2: Please clarfiy how you define 'malnutrition' here; is this all forms togehter? Wasting only, underweight only? etc. As this is an important message of the manuscript, good to know this

Line 321:

Would be good to briefly elaborate on this hypothesis. I guess you refer to bacterial translocation and a subsequent bloodstream infection? Please provide a reference here supporting this hypothesis.

Line 334: Please revise this sentence, after reading it 3x I still do not entirely understand what you mean here.

Line 340: When taking the prevalence of Adenovirus diarrhoea from GEMS, ABCD etc into account, this is a ery low prevalence. Discuss this please later and also discuss the potential pitfalls and 'subjectivity' of (the use of) a DeCoDe panel.

Line 350: I was unable to find figs 1, 2 and 3 unfortunately.

Line 373:

Table 4 comes a bit out of nowhere as in the text you are discussing figure 1-3...

Line 414:

Please be more concrete here; why is it potentially preventable? What is 'preventable' and in what setting exactly? Elaborate here

Discussion:

Line 426:

See my comment in the last paragraph of the results-section on 'potentially preventable'.

Line 436: Ok, this makes sense. But what now? Do you suggest continuing using TAC as it reveals all sorts of pathogens that are difficult to postition in the causal pathway? Describe the way forward please. Currently, this is too fact like in my opinion.

Line 449:

What I miss here is reference to the/your CHAMPS paper from 2023 (Bassat JAMA Network open) and the well written editorial by Subramanian). Have a look at the editorial and see how that paper puts MITS findings in a larger, Global Child Health perspective. That way, the (very high level and cool) data from you paper, will be taken to a higher, translational level.

Line 453: Although I agree, 'programmatic follow-up' in itself is a 'buzz word' and is not specific. These programmes have been there since at least 20 years so either these programmes do not work or needs to be changed. Tell the reader what you believe, knowing your data now, shold be done (differently). Be ambitious while concrete!

Line 462: Although not in your circle of influence, this is a limitation of the data. Pleas acknowledge that.

Line 471: Sure, but how sure are you that there are even more diarrhoeal deaths in the community that simply did not go to the hospital? Please reflect on this

Line 510: Specify here please and be concrete; what interventions and where in the 'health care pathway' should these interventions be positioned?
