# Supplementary material for: Use of minimally invasive tissue sampling to determine the contribution of diarrheal diseases to under-five mortality and associated co-morbidities and co-infections in children with fatal diarrheal diseases in Africa and Bangladesh
Source: PLOS Glob Public Health. 2025 Jun 25;5(6):e0004772. doi: 10.1371/journal.pgph.0004772 (PMC12193650; doi:10.1371/journal.pgph.0004772)
Supplement: Attachment — Submitted filename: Response_File_23May2025.docx [file pgph.0004772.s015.docx]

| Editor |  |
| --- | --- |
| EDITOR COMMENT | **AUTHOR RESPONSE** |
| Journal Requirements:  1. Please report in the Methods section the dates when data were accessed for research purposes. | We added the sentence “Data were accessed for analysis on 31 May 2024.” to the Methods section under “Statistical analyses.” Line 218 |
| Additional Editor Comments (if provided):  1. General comment on added value:  We thank the authors for their important work. As a clinician working in a referral hospital in a low- and middle-income setting, I read this paper with great interest. However, I was left with a sense of "déjà vu". It remains unclear what the real added value of this substantial effort is to the existing body of knowledge on the causes of death and diarrhea in children under five in LMICs. I recommend that the introduction and discussion be strengthened by presenting a clearer and more compelling rationale for the study. Specifically, the authors should articulate what gap this study fills and how it builds upon or differs from previous work in this area. | We appreciate the reviewer’s thoughtful feedback. We revised the Introduction and Discussion to more explicitly articulate how this study builds upon previous work. Specifically, we highlight how CHAMPS uniquely addresses the gap in understanding the causal pathways of fatal diarrhea using postmortem diagnostics and expert panel review—approaches that distinguish it from prior landmark studies like GEMS and VIDA, which examined the mortality over a 2-3 month period following an episode of microbiologically defined medically-attended diarrhea so the immediate cause of death could not be determined with certainty in many cases. MAL-ED, on the other hand, conducted community-based surveillance, detecting mostly mild cases of diarrhea for which mortality was low.  We emphasize that this study provides causally attributed, pathogen-specific mortality data—including from community deaths—and highlights the syndromic complexity and comorbidities underlying fatal cases. These additions clarify our contribution to the literature and underscore the novel insights for public health action. Lines 116-138, 444-462. |
| 2. Actionability and policy relevance of findings:  I was encouraged by the first sentence of the final paragraph: “Line 509: Our data provides valuable insights that are translatable into interventions to reduce diarrheal deaths and ultimately reduce under-five mortality.” However, I expected the authors to elaborate further on the policy and programmatic implications of their findings. How do they envision these results informing action, both locally and globally? What changes—at the level of health policy, clinical practice, or public health interventions—do the findings support? In global health, research must lead to clear, actionable recommendations to be truly impactful. | We appreciate the suggestion and agree that clarity on actionability is essential. We revised the final paragraph of the Discussion to more directly outline how our findings can inform clinical practice, vaccination policy, integrated care models, and community-based interventions. These edits clarify how CHAMPS data can drive policy and programmatic action toward achieving SDG child mortality goals. Lines 579-593. |
| 3. HIV and comorbidities:  HIV remains a major contributor to both child malnutrition and sepsis in LMICs. Did the authors examine these comorbidities—particularly HIV—either separately or in combination, and assess their interactions with diarrheal mortality? Exploring such comorbid conditions could offer additional insights, especially given the known syndemic interactions in undernourished pediatric populations. | We thank the reviewer for this suggestion. Although our dataset includes only deceased children and cannot assess mortality risk, we now explore the co-occurrence of HIV, malnutrition, sepsis, and other comorbidities among diarrhea-attributed deaths using an Upset plot (S2 Fig). This visualization shows the syndemic overlap of conditions contributing to fatal diarrhea. We also draw the reviewer’s attention to S3 Figure, which shows the directional relationships between diarrhea and other causes in the causal chain, further illustrating these interactions. |

| Reviewer #1 |  |
| --- | --- |
| REVIEWER COMMENT | **AUTHOR RESPONSE** |
| Reviewer #1: This is a well written and clear paper. I conclusion are supported by the data presented and by the conclusion of multiple antecedent studies. This manuscript offers an important lens on diarrheal mortality and will be a good addition to the literature. I have a few relatively minor comments: | Thank you for your comments. |
| The role of CT value on the qPCR was unclear to me. Were all levels of detection considered a possible cause of the diarrhea, or did the pannel consider CT value in their attribution? | Thank you for this thoughtful question. We have clarified the role of Ct values in the revised Methods (Lines 171-178). In brief, Ct values were considered by CHAMPS site laboratories and DeCoDe panels as part of a broader qualitative review, which also incorporated amplification curve shape, fluorescence intensity, assay-specific validation guidance, and clinical context. As a general practice, detections with Ct <35 were considered present, but causality was determined by panel interpretation. For some assays, including *E. coli*/Shigella, more stringent thresholds were applied (e.g., Ct >30 considered indeterminate) due to known risks of false positives [23]. Final attribution was based on the totality of evidence, including clinical, histopathologic, and epidemiologic information. |
| I do not believe that The EAEC results is in line with previous observations from GEMS and VIDA and MALED. There is a lot of EAEC around, and it is often associated with malnutrition, however it is very surprising to see it come out as the leading cause of diarrhea mortality. Is this result just confounded by nutritional status? Could this reflect a bias in the pannels mortality attribution? A richer discussion of this results is important, particularly as you have GEMS/VIDA investigators on this paper. | Thank you for the observation. We have pointed out the limitations of determining “attributable” pathogens in CHAMPS, whereas the use of matched controls in GEMS and VIDA permitted attribution (lines 550-557). To further explore the relationship of EAEC with malnutrition, we stratified diarrhea-attributed deaths by malnutrition status (S5 Table). EAEC was more frequently attributed among malnourished children but also appeared in non-malnourished deaths, suggesting nutritional status does not fully account for its prominence. We added, “we cannot exclude the possibility of attribution bias, whereby panelists may have been more likely to attribute the episode to EAEC in malnourished children than in those without malnutrition” to lines 520-523). We now discuss this result in more detail in the revised Discussion (lines 514–523) |
| Abstract: Why is Rota virus disaggregated in the abstract but no other pathogens are? Surely this just serves to demote Rota in the order of importance. It is helpful to disaggregate it in the manuscript but perhaps not in the abstract. | We thank the reviewer for this suggestion. We have revised the abstract to group rotavirus types together, consistent with how other pathogens are presented. |
| Sever wasting has been defined by WLZ – are you able to include Kwashikor and MUAC in that definition for the full SAM definition? | We thank the reviewer for this important suggestion. We have updated the manuscript to reflect a more complete definition of severe wasting by including both weight-for-length Z-scores (WLZ) and mid-upper arm circumference Z-scores (MUACZ). Specifically, we revised the Methods section to define severe wasting as WLZ < –3 SD and/or MUACZ < –3 SD, and Table 1 now includes classifications for both WLZ and MUACZ, as well as a combined “Severe wasting (WLZ or MUACZ < –3 SD)” variable.  Regarding kwashiorkor, while we recognize that its inclusion is important to fully capture all forms of SAM, systematic data on clinical signs of bilateral pitting edema were not consistently collected during CHAMPS postmortem assessments. Instead, diagnoses of kwashiorkor and related forms of protein-energy malnutrition were recorded through ICD-10/ICD-11 codes when explicitly identified by DeCoDe panels based on available data. As such, although some deaths were coded as kwashiorkor (ICD-10 E40 or ICD-11 5B50–5B54), this was not available for all decedents with anthropometric evidence of SAM, and therefore could not be consistently integrated into our definition of severe wasting. We discuss this limitation in more detail in a related CHAMPS analysis focused specifically on malnutrition (<https://gh.bmj.com/content/9/12/e017262>). |
| Line 319-325 is pretty hard to understand, but I think is making the point that children with sepsis do not die of diarrhea. This is a very convoluted way of making a point that broadly understood already. | Thank you — we’ve clarified this sentence to be more concise and interpretable, emphasizing the directional relationship between diarrhea and sepsis. Lines 332-337. |
| Line 467 says “for”, I think should be “or” | Done |

| Reviewer #2 |  |
| --- | --- |
| REVIEWER COMMENT | **AUTHOR RESPONSE** |
| ""Abstract:  Line 75: Please specify 'cause'; is this underlying, any, or immediate cause of death? | Done. |
| Intro:  Line 100: This is confusing as 2 causes can not both be the 4th leading cause. As far as I am informed, diarrhoea is the 3rd cause for <5M and malaria the 4th. Please clarfiy and change . | Thank you—we’ve clarified the ranking to reflect diarrhea as the third and malaria as the fourth leading cause of under-5 mortality per UN-IGME estimates. Lines 99-101. |
| Results:  Line 237: I am surprised by this percentage as we know from the CHAIN data and data from Uganda that close to 60% of all mortality occurs after hospital discharge, at home. Is this high% in your study a potential bias with higher illness severity? | Thank you. We agree this is an important point and have clarified in the limitations that CHAMPS data are biased toward facility-based deaths due to logistical constraints around postmortem enrollment, including timing of death and consent for MITS. Lines 554-559. |
| Line 246: Bangladesh seems an outlier in the study; in numbers, %wasted and % underweight. Happy to see this discussed somewhere. | Thank you for noting this. We now mention Bangladesh’s distinct profile in the Results, and note its small sample size in the limitations. Lines 265-266, 561-562. |
| Line 286:  It would be important to know if these children, vaccinated for Rotavirus, where indeed Rota+ve. This then might have been a vaccine failure? Please elaborate and clarify. | Thank you for this helpful comment. We have clarified in the text that 8 of the 32 deaths attributed to rotavirus had documentation of rotavirus vaccination. However, the timing of infection relative to vaccination is unknown. These cases may reflect primary vaccine failure, infection before completing the vaccine series, or insufficient time for immunity to develop—rather than waning immunity, which is less likely given the young age of these children. |
| Line 290:  This seems an important missing item as in 432 children, information on Rotavirus vaccination is missing. I think that currently 'but rather limitations in accessing complete immunization data', is underestimating this lack of data. Please amend this sentence, showing this. | We agree and revised the sentence to better reflect the magnitude of missing data—190 of 240 cases were either unvaccinated or had unknown status—highlighting both incomplete coverage and documentation challenges. Lines 300-305. |
| Line 297:  This is likely a reflection of the fact that many deaths happened in the hospital with sicker children. If you have more information on co-morbidities and signs of illness severity, good to show them. That way, the reader will have a btter understanding of the clinical state these children were in while admitted. | We thank the reviewer for this suggestion. We have added a sentence specifying the most common co-morbidities (malnutrition, sepsis, lower respiratory infections, anemia, and HIV) among children with diarrhea deaths who died in healthcare facilities, and referenced Table 3 and S2 Fig for additional detail (Lines 323-324). |
| Line 307: You must know if the pathogens/pathogen profile in the groups where diarrhoea is underlying versus immediate is different. It is not unlikely that both groups (also because of the immunodeficiency caused by the malnutrition) leads to different pathogens causing the diarrhoea. Do you have information of children with no pathogen while being malnourished; as in malnutrition-enteropathy leading to death? | We appreciate this insightful comment. As noted in the Results (Lines 390–391), pathogen profiles were broadly similar when stratified by whether diarrhea was listed as the underlying versus immediate/antecedent cause of death (Fig 2). To address potential differences by nutritional or immunologic status, we added a supplemental table (S5 Table) showing pathogens attributed to diarrheal deaths, stratified by malnutrition, HIV infection, and absence of both conditions. We also clarify in the Results that 42.0% of diarrhea deaths without a specific pathogen attributed had malnutrition listed in the causal pathway (S4 Table). As discussed (Lines 527–529), many of these children had enteric pathogens detected by PCR, suggesting that malnutrition-associated enteropathy or competing comorbidities may have influenced attribution decisions. |
| Line 307-2: Please clarfiy how you define 'malnutrition' here; is this all forms togehter? Wasting only, underweight only? etc. As this is an important message of the manuscript, good to know this | We thank the reviewer for this helpful suggestion. We have clarified in the Methods section that malnutrition refers to DeCoDe panel attribution based on clinical, anthropometric, and postmortem data and may include underweight, wasting, stunting, or combinations thereof (Lines 188-191). |
| Line 321:  Would be good to briefly elaborate on this hypothesis. I guess you refer to bacterial translocation and a subsequent bloodstream infection? Please provide a reference here supporting this hypothesis. | Thank you for your comment. We revised the language to more neutrally describe the observed relationship between diarrhea and sepsis attribution without implying a specific mechanistic hypothesis (Line 332-337) |
| Line 334: Please revise this sentence, after reading it 3x I still do not entirely understand what you mean here. | We thank the reviewer for this suggestion. We have revised the sentence for clarity to better explain that many diarrhea deaths without an attributed pathogen still had pathogens detected by TAC PCR (Lines 346-348) |
| Line 340: When taking the prevalence of Adenovirus diarrhoea from GEMS, ABCD etc into account, this is a ery low prevalence. Discuss this please later and also discuss the potential pitfalls and 'subjectivity' of (the use of) a DeCoDe panel. | Thank you--we have added text to the Discussion noting the lower adenovirus attribution compared to studies like GEMS, and we discuss both potential reasons for this difference and the strengths and limitations of the DeCoDe panel approach (Lines 507-512) |
| Line 350: I was unable to find figs 1, 2 and 3 unfortunately. | We thank the reviewer for noting this. Figures 1, 2, and 3 were uploaded with the original submission and have been reuploaded with this revision. |
| Line 373:  Table 4 comes a bit out of nowhere as in the text you are discussing figure 1-3... | We thank the reviewer for the comment. Per PLOS formatting guidelines, we cite figures and tables in ascending numeric order at first appearance. As required, Table 4 is cited upon first mention in the text and placed immediately afterward. |
| Line 414:  Please be more concrete here; why is it potentially preventable? What is 'preventable' and in what setting exactly? Elaborate here | We have added clarification to the Methods section. Preventability was determined by DeCoDe panels based on conditions immediately surrounding each death, using all available clinical, pathological, and contextual information, and did not extend to broader societal factors. We also clarified that for preventable deaths, specific health system gaps were identified and categorized into key domains such as clinical management, health education, and health-seeking behavior (Lines 196-202). We also revised the Results (Lines 429-434) to more concretely describe why deaths were considered preventable, including examples of the most frequently identified gaps in care. |
| Discussion:  Line 426:  See my comment in the last paragraph of the results-section on 'potentially preventable'. | We clarified the definition of “potentially preventable” in the Methods and revised the Discussion to specify that preventability was based on identifiable health system gaps (Lines 440-444). |
| Line 436: Ok, this makes sense. But what now? Do you suggest continuing using TAC as it reveals all sorts of pathogens that are difficult to postition in the causal pathway? Describe the way forward please. Currently, this is too fact like in my opinion. | Thank you for this helpful suggestion. In response, we added text to the Discussion (Lines 472–475) describing future approaches to improve causal attribution—including the use of pathogen quantity thresholds, integration with clinical and histopathologic evidence, and the incorporation of control groups to distinguish infection from colonization. |
| Line 449:  What I miss here is reference to the/your CHAMPS paper from 2023 (Bassat JAMA Network open) and the well written editorial by Subramanian). Have a look at the editorial and see how that paper puts MITS findings in a larger, Global Child Health perspective. That way, the (very high level and cool) data from you paper, will be taken to a higher, translational level. | Thank you for this suggestion. We have now cited both the Bassat et al. (2023) CHAMPS paper and the accompanying editorial by Subramanian to situate our findings in a broader, translational, global child health context (Lines 492-497) |
| Line 453: Although I agree, 'programmatic follow-up' in itself is a 'buzz word' and is not specific. These programmes have been there since at least 20 years so either these programmes do not work or needs to be changed. Tell the reader what you believe, knowing your data now, shold be done (differently). Be ambitious while concrete! | Thank you for this suggestion. We revised the Discussion to replace "programmatic follow-up" with specific actions, including routine screening, HIV testing, longitudinal follow-up, and early targeted interventions (Lines 497-500). |
| Line 462: Although not in your circle of influence, this is a limitation of the data. Pleas acknowledge that. | Thank you for the suggestion. We have added a statement acknowledging incomplete vaccination records as a limitation (Lines 508-509) |
| Line 471: Sure, but how sure are you that there are even more diarrhoeal deaths in the community that simply did not go to the hospital? Please reflect on this | Thank you for the comment. We have reflected on this point by noting that we cannot definitively assess whether more diarrheal deaths occurred in the community without reaching care, given limitations in death capture and enrollment (Lines 537-539) |
| Line 510: Specify here please and be concrete; what interventions and where in the 'health care pathway' should these interventions be positioned?"" | Thank you for this suggestion. We revised the paragraph to specify concrete interventions—such as rehydration, antimicrobial stewardship, nutritional support, and community case detection—and clearly positioned these along the healthcare pathway, including facility-based care, integrated management of comorbidities, and community-level prevention (Lines 580–588). |
